# Updates on Vision From the Global Burden of Disease Study

**DOI:** 10.1093/geroni/igaa057.2935

**Published:** 2020-12-16

**Authors:** Jaimie Adelson, Paul Briant, Seth Flaxman, Hugh Taylor, Serge Resnikoff, Nikolas Reinig, Rupert Bourne, Theo Vos

**Affiliations:** 1 University of Washington, Seattle, Washington, United States; 2 Institute for Health Metrics and Evaluation, Seattle, Washington, United States; 3 Imperial College London, London, England, United Kingdom; 4 Melbourne School of Population Health, Melbourne, Victoria, Australia; 5 Brien Holden Vision Institute, Sydney, New South Wales, Australia; 6 Vision and Eye Research Institute, Cambridge, England, United Kingdom

## Abstract

The Global Burden of Disease study estimates the prevalence of vision loss by WHO severity group, and the distribution of vision loss due to underlying causes such as cataract, glaucoma, and uncorrected refractive error. Data sources include national and local surveys, and scientific literature, with over 500 sources in 113 countries. Cause-specific prevalence estimates were proportionally fit into estimates of total vision loss by severity. Globally, 821.9 million (95% UI=682.2–970.4) people experienced moderate or worse distance vision loss or near vision loss in 2019 (age-standardized prevalence = 0.10, UI=0.08–0.12). Of these, 41.9 million (UI=36.4–46.8) were blind. The majority of Years Lived with Disability due to vision loss occurred in people with cataract and uncorrected refractive error. Vision loss numbers will continue to increase due to aging populations. Main causes of vision loss are correctable and, therefore, should be major targets of interventions.

